# Total lesion glycolysis of primary tumor and lymphnodes is a strong predictor for development of distant metastases in oropharyngeal carcinoma patients with independent validation in automatically delineated lesions

**DOI:** 10.1186/s40644-025-00836-6

**Published:** 2025-02-21

**Authors:** Sebastian Zschaeck, Marina Hajiyianni, Patrick Hausmann, Pavel Nikulin, Emily Kukuk, Christian Furth, Paulina Cegla, Elia Lombardo, Joanna Kazmierska, Adrien Holzgreve, Iosif Strouthos, Carmen Stromberger, Claus Belka, Michael Baumann, Mechthild Krause, Guillaume Landry, Witold Cholewinski, Jorg Kotzerke, Daniel Zips, Jörg van den Hoff, Frank Hofheinz

**Affiliations:** 1 Department of Radiation Oncology, Charité– Universitätsmedizin Berlin, corporate member of Freie Universität Berlin, Humboldt-Universität zu Berlin, Berlin Institute of Health, Augustenburger Platz 1, 13353 Berlin, Germany; 2 Berlin Institute of Health at Charité, Universitätsmedizin Berlin, BIH Biomedical Innovation Academy, BIH Charité (Junior) Clinician Scientist Program, Berlin, Germany; 3 Department of Radiotherapy and Radiation Oncology, Faculty of Medicine and University Hospital Carl Gustav Carus, Technische Universität Dresden, Dresden, Germany; 4 German Cancer Consortium (DKTK), partner site Dresden, Germany, and German Cancer Research Center (DKFZ) Heidelberg, Heidelberg, Germany; 5 OncoRay– National Center for Radiation Research in Oncology, Faculty of Medicine and University Hospital Carl Gustav Carus, Technische Universität Dresden, Helmholtz-Zentrum Dresden– Rossendorf, Dresden, Germany; 6 Institute of Radiopharmaceutical Cancer Research, Helmholtz-Zentrum Dresden-Rossendorf, Dresden, Germany; 7 Department of Nuclear Medicine, Charité Universitätsmedizin Berlin, Freie Universität Berlin and Humboldt Universität zu Berlin, Berlin Institute of Health, Berlin, Germany; 8 Department of Nuclear Medicine, Greater Poland Cancer Centre, Poznan, Poland; 9 Department of Radiation Oncology, LMU University Hospital, LMU Munich, Munich, Germany; 10 Electroradiology Department, University of Medical Sciences, Poznan, Poland; 11 Radiotherapy Department II, Greater Poland Cancer Centre, Poznan, Poland; 12 Department of Nuclear Medicine, LMU University Hospital, LMU Munich, Munich, Germany; 13 Department of Radiation Oncology, German Oncology Center, European University Cyprus, Limassol, Cyprus; 14 Bavarian Cancer Research Center (BZKF), Munich, Germany; 15 German Cancer Consortium (DKTK), partner site Munich, a partnership between DKFZ and LMU University Hospital Munich, Munich, Germany; 16 Division of Radiooncology/Radiobiology, German Cancer Research Center (DKFZ), Heidelberg, Germany; 17 German Cancer Consortium (DKTK), partner site Munich, a partnership between DKFZ and LMU University Hospital Munich, Heidelberg, Germany; 18 Faculty of Medicine and University Hospital Carl Gustav Carus, Technische Universität Dresden, Dresden, Germany; 19 Helmholtz Association/Helmholtz-Zentrum Dresden - Rossendorf (HZDR), Dresden, Germany; 20 Institute of Radiooncology, Helmholtz-Zentrum Dresden-Rossendorf, Dresden, Germany; 21 Department of Nuclear Medicine, University Hospital Carl Gustav Carus, Technische Universität Dresden, Dresden, Germany; 22 German Cancer Consortium (DKTK), partner site Berlin, a partnership between DKFZ and Charité– Universitätsmedizin Berlin, Berlin, Germany; 23 National Tumor Center Berlin (NCT), Partner Site Berlin, Germany: German Cancer Research Center (DKFZ), Heidelberg, Germany; 24 Charité Comprehensive Cancer, Charité Universitätsmedizin Berlin, Berlin, Germany; 25 Berlin Institute of Health, Charité Universitätsmedizin Berlin, Berlin, Germany; 26 Max-Delbrück-Centrum für Molekulare Medizin, Helmholtz Association, Berlin, Germany

**Keywords:** Head and neck squamous cell carcinoma, Fluorodeoxyglucose positron emission tomography, Oropharynx cancer, Total lesion glycolysis

## Abstract

**Background:**

Oropharyngeal carcinomas are characterized by an increasing incidence and a relatively good prognosis. Nonetheless, a considerable number of patients develops metachronous distant metastases; identification of these patients is an urgent medical need.

**Methods:**

This is a retrospective multicenter evaluation of 431 patients. All patients underwent [^18^F]-FDG positron emission tomography (PET). The cohort was split into an explorative group (*n* = 366) and a validation group (*n* = 65). Lesions were manually delineated in the explorative group and automatically delineated by a convolutional neuronal network (CNN) in the validation group. Quantitative PET parameters standardized uptake value (SUV), metabolic tumor volume (MTV), and total lesion glycolysis (TLG) were calculated for primary tumors (_prim_) and tumor plus lymphnodes (_all_). Association of parameters with freedom from distant metastases (FFDM) and overall survival (OS) was tested by cox regression analyses.

**Results:**

In the explorative group, univariate analyses revealed an association of metric MTV_prim_ (*p* = 0.022), MTV_all_ (*p* < 0.001) and TLG_all_ (*p* < 0.001) with FFDM, binarized parameters were also associated with FFDM (*p* < 0.001 and *p* = 0.002). Bootstrap analyses revealed a significantly better association of TLG_all_ compared to TLG_prim_ with FFDM (*p* = 0.02). MTV_all_ and TLG_all_ remained significantly associated with FFDM upon multivariate testing (*p* = 0.002, *p* = 0.031, respectively). In the validation group, the cutoff value for TLG_all_ but not for TLG_prim_ was significantly associated with FFDM (HR = 3.1, *p* = 0.045). Additional analyses with manually delineated contours of the validation cohort revealed a similar effect (HR = 3.47, *p* = 0.026). No considerable differences between HPV positive and negative disease were observed.

**Conclusions:**

TLG_all_ is a promising biomarker to select OPC patients with high risk for metachronous distant metastases.

**Supplementary Information:**

The online version contains supplementary material available at 10.1186/s40644-025-00836-6.

## Background

Oropharyngeal squamous cell carcinoma (OPC) is the most prevalent head and neck cancer site in Western countries. Due to its frequent association with prior human papilloma virus (HPV) infection, incidence of this subtype is still increasing in contrast to a decreasing incidence of tobacco induced tumors at other head and neck sites [1–3]. HPV associated OPC is characterized by a favorable prognosis and high radiosensitivity [4]. Depending on tumor stage, local control rates range between 80% and 100% [4, 5]. Due to the high loco-regional control rates, even in locally advanced disease, distant metastases become more clinically relevant. There is some evidence that HPV positive tumors respond more favorably to checkpoint inhibition [6]. Given the negative clinical trials on addition of checkpoint inhibition to radiotherapy / chemoradiation (CRT), it is a clinically highly relevant issue to detect patients with high risk for metachronous metastases before initial treatment [7, 8]. These patients would be candidates for additional intensified treatment during or after CRT, for example consolidation checkpoint inhibition that has been shown to be highly effective in lung cancer [9].

Positron emission tomography (PET) staging with the radiotracer 18 F-flurodeoxyglucose (FDG) is standard of care for locally advanced OPC that is treated with definitive CRT [10]. Routinely obtained PET parameters can be used as quantitative imaging biomarkers, that can potentially be used for patient stratification. Commonly, these parameters are only evaluated within the primary tumor since delineation of tumor and affected lymph nodes is labour intensive and might be prone to interobserver bias. We have developed a convolutional neuronal network (CNN) to automatically delineate the PET signal of affected head and neck lymph nodes [11]. We hypothesized that inclusion of regional lymph nodes improves the ability of quantitative PET parameters to predict distant metastases. Furthermore, the prognostic value was validated in an independent cohort, that was fully automatically delineated by the previously published CNN to ensure fast and reproducible evaluation of this biomarker.

## Methods

### Patients and data acquisition

Altogether 431 patients (96 women, 335 men, mean age 61 ± 9y) from three public databases (The Cancer Imaging Archive) and five European centers were included in this study [12–17]. These patients were a subgroup of the patient group already published in [18], where only the primary tumor was investigated. Inclusion criteria in this study were: histologically confirmed head and neck squamous cell carcinoma (HNSCC) without evidence of distant metastases, definitive radiotherapy or CRT with curative intent, and availability of an 18 F-FDG- PET prior to therapy. Additional inclusion criteria in the present analysis were: oropharyngeal primary tumor location and availability of a corresponding computed tomography (CT) scan, information on development of distant metastases, and sufficient information to compute standardized uptake values (SUV). In eight cases not all PET positive lymph nodes were fully in the field of view. These eight patients were excluded for further analyses. A summary of patient and tumor characteristics is given in Table 1. Note that HPV status was available only for about half of the patients. Data acquisition was performed with eight different PET/CT systems (see [18]) and started 88 ± 19 min after injection of 262 to 483 MBq 18 F-FDG. Tomographic images were reconstructed using the standard reconstruction of the corresponding PET/CT system.

**Table 1:** Patient and tumor characteristics

Characteristics	Value
**Age (years)**	
Mean ±SD Median	61 ± 9 61
**Sex**	
Male	335 (77.7)
Female	96 (22.3)
**T stage**	
Tx	1 (0.2)
T1	50 (11.6)
T2	143 (33.2)
T3	118 (27.4)
T4	117 (27.1)
**N stage**	
N0	56 (13)
N1	51 (11.8)
N2	295 (68.4)
N3	25 (5.8)
**UICC stage**	
I	4 (0.9)
II	23 (5.3)
III	63 (14.6)
IV	341 (79.1)
**HPV status**	
n/a	219 (50.8)
negative	89 (20.6)
positive	123 (28.5)

### Treatment

All patients received primary radiotherapy with curative intent, performed as three-dimensional, intensity modulated or volumetric modulated treatment. Prescribed radiation doses ranged between 66 and 77 Gy (Gy). 366 patients received concomitant systemic therapy, 63 patients received radiotherapy only and 2 patients had no information available on concomitant therapy.

### Image analysis

For analysis the patient sample was split into an explorative group (*N* = 366) and a validation group (*N* = 65). The reason for this explicit splitting is based on the training data of the CNN for automatic delineation and explained in detail below. Distribution of clinical parameters were comparable in both groups.

In the explorative group the metabolically active part of the primary tumor and of all PET positive lymph node metastases was delineated in the PET data by an automatic algorithm based on adaptive thresholding considering the local background [19, 20]. Affected lymphnodes were selected based on FDG uptake or CT parameters. The resulting region of interest (ROI) delineation was inspected visually by an experienced observer (who was blinded to patient outcome) and manually corrected when this was deemed necessary. This was the case in 14 out of 366 primary tumors. Manual correction was more frequent for the lymph nodes: 248 out of 621 lesions. In all cases the reason for manual intervention was a low diffuse tracer accumulation in the respective lesion.

In the validation group the lesions were delineated by application of a previously published CNN [11]. This network was trained and tested with the data used in [18], which includes the data analyzed in this work. In [11] the CNN was tested with external data, i.e. data which had not been used during the training process. The validation group data consists of all data from this independent sample, matching the above mentioned inclusion criteria (availability of CT as well as SUV and oropharyngeal tumor location). CNN based delineation was reviewed but not corrected, resulting in an observer independent delineation. Manually delineated contours were also available for the validation cohort and used for additional validation of robustness. Note that a CNN based delineation of the lesions in the explorative group would not lead to an observer independent delineation. All data in this group were used for CNN training. Application of the CNN to these data would, therefore, introduce a bias towards the delineating observer. Details on the implementation of the CNN to the current data can be found in supplementary methods 1.

For all delineated ROIs, the parameters maximum and mean SUV (SUV_mean_ and SUV_max_), metabolic tumor volume (MTV), and total lesion glycolysis (TLG = MTV×SUV_mean_) were calculated. All three parameters were computed for the primary tumor alone and for primary tumor plus lymph node metastases. In the following we refer to the parameters derived from the primary tumor as SUV_prim_, MTV_prim_, and TLG_prim_, where SUV_prim_ is the SUV_max_ of the primary tumor. The parameters derived from all lesions were denoted as SUV_all_, MTV_all_, and TLG_all_, where SUV_all_ is the overall SUV_max_, MTV_all_ is the cumulative volume of primary tumor and lymph nodes, and TLG_all_ is the overall SUV_mean_ times MTV_all_.

ROI definition and analysis was performed using the ROVER software, version 3.0.47 (ABX, Radeberg, Germany).

### Statistical analysis

The investigated clinical endpoint was freedom from distant metastases (FFDM) measured from the start of therapy to death and/or event. The association of FFDM with clinically relevant parameters (age, sex, T-stage, N-stage, UICC-stage, chemotherapy yes/no, and HPV status) as well as quantitative PET parameters derived in the explorative group was analyzed using univariate Cox proportional hazard regression in which the PET parameters were included as metric parameters. PET parameters showing a significant effect (*P* < 0.05) or a trend for significance (*P* < 0.1) in this analysis were further analyzed in univariate Cox regression using binarized PET parameters. The cutoff values used for binarization were calculated by performing a univariate Cox regression for each measured value. The values leading to the hazard ratio (HR) with the highest significance were used as cutoff. Hazard ratios were compared using the bootstrap method (10^5^ samples) to determine the statistical distribution of (HR1 − HR2) from which the relevant *P*-value then was derived. In case of a significant difference in HR between parameters derived from the primary tumor and parameters derived from all lesions the corresponding cutoff values were applied to the data in the validation group. The probability of survival was computed and rendered as Kaplan-Meier curves. Independence of parameters was analyzed by multivariate Cox regression, where the clinical parameters, which showed a significant effect in univariate analysis, were included as confounding factors.

Statistical significance was assumed at a *P*-value of less than 0.05. Statistical analysis was performed with the R language and environment for statistical computing version 4.2.2 [21].

## Results

Patient characteristics of the whole cohort of patients are summarized in Table 1, patients were predominantly male and had stage IV disease without evidence of distant metastases in PET staging. According to our previous publication on CNN based automatic lymph node segmentation all patients were grouped accordingly. All patients whose data have been used for CNN training of lymph node segmentation, i.e. patients with manually delineated lymphnodes, were used as a training cohort (*n* = 366). For independent validation, patients of the CNN validation cohort were used (*n* = 65).

### Explorative analysis

**Fig. 1:**
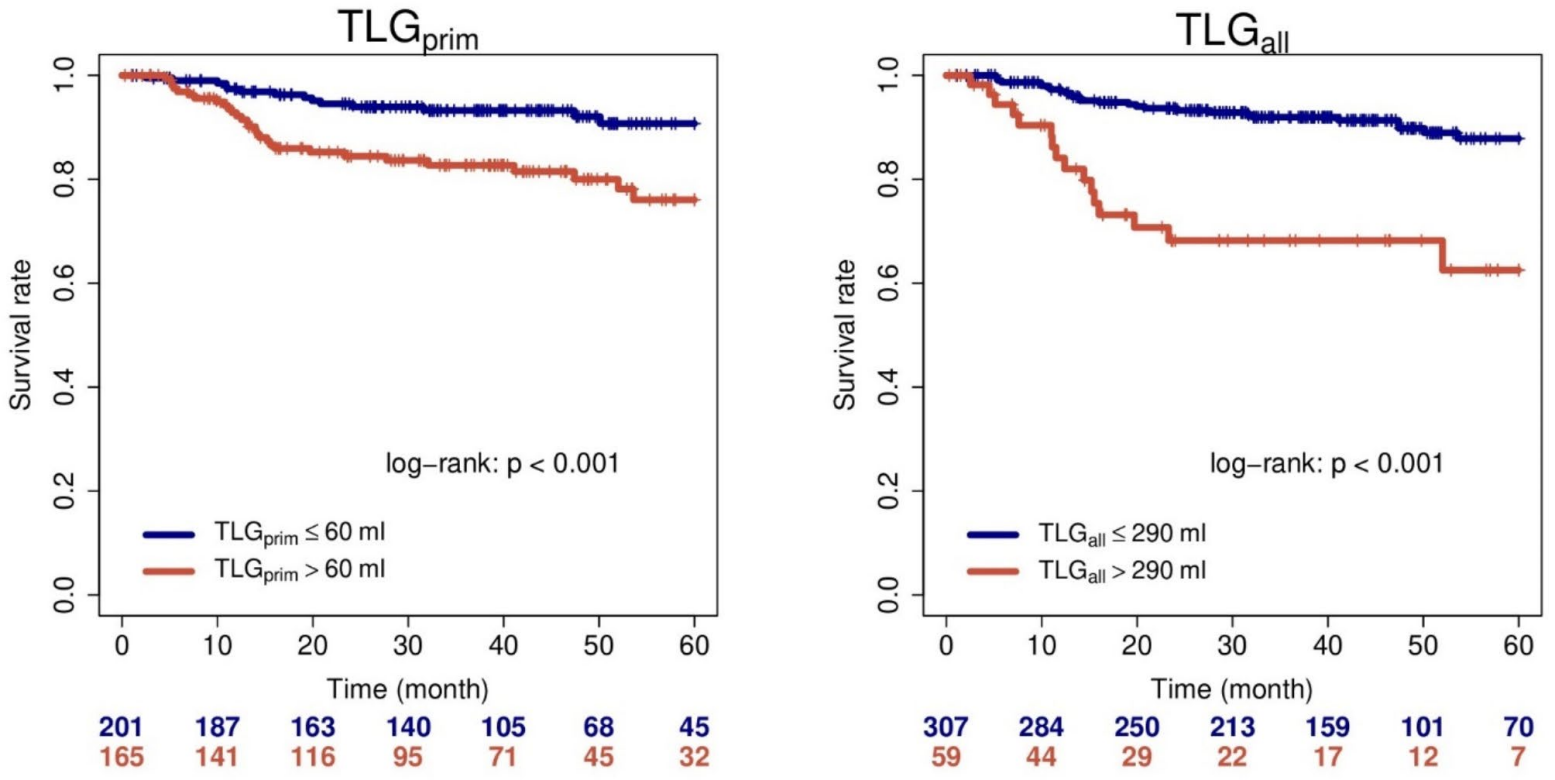
Kaplan-Meier curves with respect to FFDM. Results for the explorative group, cutoff values were optimized for this group

Univariate Cox regression using metric PET parameters revealed MTV_prim_, MTV_all_, and TLG_all_ as statistically significant prognostic factors for FFDM. TLG_prim_ showed a statistical trend for significance. For SUV_prim_ and SUV_all_ no significant statistical association with FFDM was observed. Both parameters were therefore excluded from further analyses. Among the investigated clinical parameters only T-stage was a statistical significant prognostic factor. The results for all parameters are shown in Table 2. All statistically significant metric PET parameters were binarized as described above. In univariate Cox regression the binarized parameters remained statistically significantly associated with the investigated clinical endpoints. Corresponding HRs as well as applied cutoff values can be found in Table 3. Figure 1 shows Kaplan Meier estimates for TLG_prim_ and TLG_all_ and the endpoint FFDM in the explorative cohort.

**Table 2:** Univariate Cox regression with respect to FFDM. PET parameters were included as metric parameters

Parameter	HR	95% CI	*P*-value
Sex male	2.18	0.86–5.54	0.1
Age *>* 61y	1.49	0.81–2.72	0.2
T-stage *>* 2	2.65	1.38–5.09	**0.003**
N-stage *>* 1	1.8	0.8–4.04	0.16
UICC-stage *>* III	1.7	0.72–4.04	0.23
Chemotherapy none	0.96	0.43–2.16	0.92
HPV negative	1.48	0.55–4	0.44
MTV_prim_	1.03	1–1.05	**0.022**
MTVall	1.04	1.02–1.06	*<* **0.001**
TLG_prim_	1.001	1–1.003	**0.084**
TLGall	1.002	1.001–1.004	*<* **0.001**
SUV_prim_	1.02	0.98–1.06	0.33
SUVall	1.03	0.99–1.08	0.11

**Table 3:** Univariate Cox regression with respect to FFDM. PET parameters were included as binarized parameters

Parameter	Risk	HR	95% CI	*P* value
MTV_prim_	*>* 16 ml	2.86	1.56–5.25	*<* **0.001**
MTV_all_	*>* 19 ml	4.28	2.23–8.2	*<* **0.001**
TLG_prim_	*>* 60 ml	2.8	1.48–5.31	**0.002**
TLG_all_	*>* 290 ml	4.32	2.32–8.03	*<* **0.001**

To test if inclusion of lymph nodes led to a significant improvement of the prognostic value compared to evaluation of the primary tumor alone, a pairwise bootstrap analysis was performed as described above. In this analysis TLG_all_ showed a statistically significantly increased HR compared to its primary tumor counterpart (*p* = 0.02). MTV_all_ showed only a statistical trend for significance when compared to MTV_prim_ (*p* = 0.09).

All four PET parameters (MTV_prim_, MTV_all_, TLG_prim_ and TLG_all_) showed a statistically significant association with OS, with all *p* values ≤ 0.001, see supplementary Table 1.

Multivariate Cox regression was performed with the statistically significant PET parameters and T-stage (the only significant clinical parameter) for the endpoint FFDM. However, due the strong correlation of MTV_prim_, MTV_all_, TLG_prim_, and TLG_all_ (R^2^ > 0.8) a separate analysis of each of these four parameters together with T-stage was performed. This analysis revealed MTV_all_ and TLG_all_ as statistically significant (*p* = 0.002, *p* = 0.031, respectively) factors. No statistically significant effect was found for MTV_prim_ and TLG_prim_ (*p* = 0.58 and *p* = 0.87, respectively).

### Validation

**Fig. 2:**
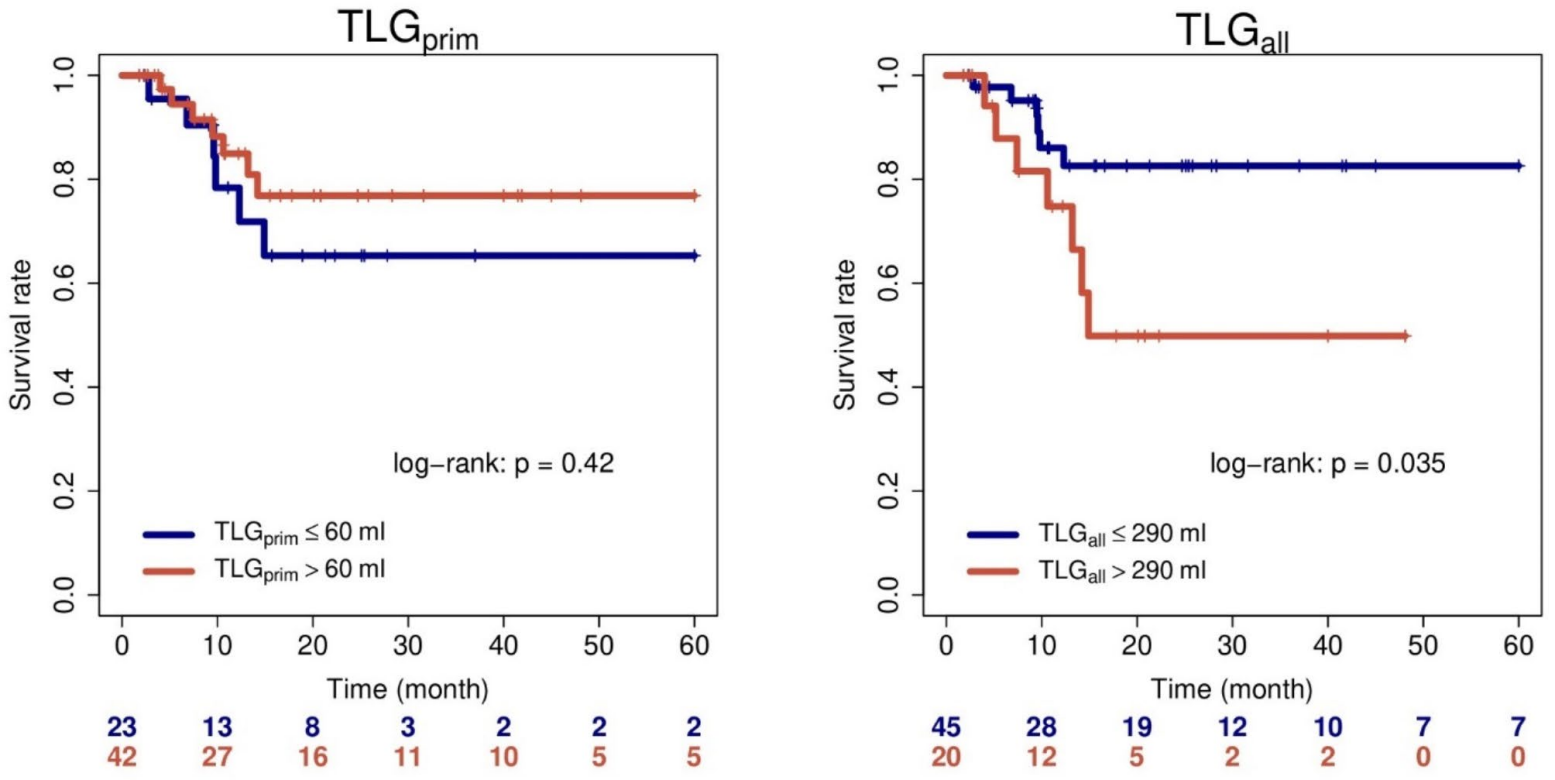
Kaplan-Meier curves with respect to FFDM. Results for the validation group, cutoff values were taken from the explorative group

The optimal cutoff values for TLG_prim_ and TLG_all_, which were found in the explorative group, were applied the data in the validation group. This revealed a statistically significant effect in univariate Cox regression for TLG_all_ (HR = 3.1, *p* = 0.045). TLG_prim_ of the primary tumor alone was not significantly associated with FFDM in this patient group (*p* = 0.42). Corresponding Kaplan-Meier curves for the validation cohort are shown in Fig. 2. Additional analyses with manually delineated contours of the validation cohort revealed a similar effect (TLG_all_ HR = 3.47, *p* = 0.026), see supplementary Fig. 1.

### HPV subgroup analyses

HPV status was available only for about 50% of the patients. Therefore, a subgroup analysis in HPV positive and negative patients was performed in all available patient data (explorative and validation cohort). Due to the strongly reduced number of patients and the relatively small number of events for the given endpoint we included as many patients as possible in this analysis. Therefore, for evaluation of MTV_prim_ and MTV_all_ also patients without information on SUV were included, which were excluded in direct comparison of different PET parameters to avoid bias by different numbers of patients (i.e. larger number of patients with MTV versus lower numbers with SUV). By extending the analyzed group of patients we were able to include 251 instead of 161 patients in this analysis. While an analysis in the explorative group alone would have been possible in principle, the validation group would have been too small for this analysis. Note that due to this extension the results below are not validated independently.

Subgroup analyses of patients with known HPV status revealed a similar performance of TLG_all_ and MTV_all_ in HPV positive and negative patients. In 141 patients with HPV positive OPC, univariate metric cox regression analyses of MTV_prim_ and TLG_prim_ only showed a statistical trend for association with FFDM (*p* = 0.098 and *p* = 0.054, respectively), while MTV_all_ (*p* < 0.001) and TLG_all_ (*p* > 0.001) were significantly associated with FFDM. Effects were maintained after binarization and in multivariate testing. Supplementary Fig. 2 shows Kaplan Meier estimates for HPV positive patients.

In 110 patients with HPV negative OPC, similar associations were seen. While MTV_prim_ and TLG_prim_ did not show any association with FFDM in metric univariate cox regression analyses, both MTV_all_ (*p* = 0.016) and TLG_all_ (p 0.035) showed a statistically significant association with outcome. The effect was maintained after binarization. No multivariate testing was performed since there were no clinical parameters associated with FFDM in this small sub-group. Supplementary Fig. 3 shows Kaplan Meier estimates for HPV negative patients.

## Discussion

Here we were able to show that the semi-quantitative PET parameter TLG_all_ was able to stratify patients with a high risk for metachronous distant metastases. This was only the case if primary tumor and all affected lymph nodes were included in the analyses; evaluation of the primary tumor alone did not remain statistically significantly associated with outcome upon independent validation. Importantly, validation was performed in a fully automatically segmented independent group of patients and yielded a significant discrimination of risk groups similar to manually segmented contours of the validation group. We think that this is a very important prerequisite for clinical implementation, since it avoids inter-observer variability and reduces the workload of dedicated physicians.

Prediction of patients at high risk of developing distant metastases during follow-up is an urgent medical need and several publications have developed methods for outcome prediction in HNSCC patients. Wang and colleagues developed a deep learning based model that was able to predict distant metastases in a cohort of 477 head and neck cancer patients. They investigated combined models using CT and PET scans and found out that PET information alone obtains the best model for outcome prediction [22]. Ma and colleagues compared several methodological approaches in CT images of OPC patients for outcome prediction [23]. Different models showed promising performance to predict distant metastases. Nonetheless, what both models share in common is that they seem to be better in identifying low risk patients who are unlikely to develop distant metastases. Identification of the relatively few high-risk patients seems to be a more difficult, nonetheless clinically very important task. This is a strength of our model that it was able to identify the group of patients with a very high probability (40–50%) to develop distant metastases during follow up.

It is surprising that the inclusion of affected lymph nodes only significantly improved FFDM prediction when using TLG, but not when using MTV. This might be due to the sample size of this study, which is nonetheless relatively large compared with other publications on the predictive power of PET for HNSCC outcomes. Therefore, TLG of all lesions might be a better parameter to predict the risk for distant metastases. There is less data on the association of outcome and TLG compared to MTV in HNSCC. A recent review revealed that both MTV and TLG of the primary tumor were associated with OS and disease-free survival (DFS) of HNSCC patients [24]. Interestingly, ten studies were included that investigated MTV and DFS, while only five studies reported data on TLG and DFS, and similar disparities existed for the endpoint OS. In a small study on 46 patients with locally advanced laryngeal cancer, TLG of the primary tumor was statistically significantly associated with OS and distant metastases free survival [25].

Our results are in contrast to a recent retrospective study of 57 patients with HPV associated OPC by Noor and colleagues. They reported a statistical significant association of primary tumor MTV and TLG with DFS and OS. However, nodal and total MTV and TLG did not show an association with outcome of patients [26]. This might be an accidental finding since the cohort of Noor was relatively small and the number of events is low in HPV associated tumors. Similar to our findings, Floberg and colleagues reported a significant association of total MTV of all lesions with FFDM but no significant statistical association of primary tumor MTV and FFDM in 153 patients with HPV positive OPC [27]. Corresponding TLG data was unfortunately not reported in this study. Data on TLG was reported in another study by Chotchutipan and colleagues that evaluated 142 patients with HPV related OPC. In their analyses, both MTV_all_ and TLG_all_ were statistically significantly associated with FFDM and remained significantly associated after multivariate testing [28]. In that study no direct comparison between both imaging biomarkers was performed, but univariate hazard ratios of TLG were slightly higher than for MTV. Taken together this data is quite similar to our findings in a larger cohort with both HPV positive and negative disease.

Our study has several strengths: To our best knowledge, this is the first study that systematically compared the prognostic value of the primary tumor with the prognostic value of quantitative parameters of all detectable lesions. Additionally, it includes an independent validation cohort that was automatically delineated and could be easily implemented into a clinical workflow for biomarker quantification purposes. Nonetheless, our study also has some limitations, which are mostly inherent to retrospective analyses. This includes missing information on treatment details and follow-up of some patients, missing information on HPV status in several patients and heterogeneity of treatment approaches. Although it is important to note that we tried to minimize this by excluding patients with primary surgical treatment or without sufficient information on distant metastases. The missing HPV status of patients led to very small sub-groups, therefore these results have to be interpreted extremely cautious.

## Conclusions

In summary, our data identified TLG of primary tumor and affected lymph nodes as a risk factor for the development of metachronous distant metastases in OPC patients treated with primary RT/CRT. We think that this finding merits further prospective validation, as it could potentially be very useful to personalize individual consolidation treatment of high-risk patients.

## Electronic supplementary material

Below is the link to the electronic supplementary material.

Supplementary Material 1

## Data Availability

The datasets generated during and/or analyzed during the current study are available from the corresponding author on reasonable request.

## Abbreviations

CNN: Convolutional neuronal network
CRT: Radiotherapy / chemoradiation
CT: Computed tomography
FDG: 18 F-flurodeoxyglucose
FFDM: Freedom from distant metastases
Gy: Gray
HNSCC: Head and neck squamous cell carcinoma
HPV: Human papilloma virus
HR: Hazard ratio
MTV: Metabolic tumor volume
OPC: Oropharyngeal squamous cell carcinoma
OS: Overall survival
PET: Positron emission tomography
ROI: Region of interest
SUV: Standardized uptake values
TLG: Total lesion glycolysis
