# Correction: Total lesion Glycolysis of primary tumor and lymphnodes is a strong predictor for development of distant metastases in oropharyngeal carcinoma patients with independent validation in automatically delineated lesions

**DOI:** 10.1186/s40644-025-00870-4

**Published:** 2025-04-07

**Authors:** Sebastian Zschaeck, Marina Hajiyianni, Patrick Hausmann, Pavel Nikulin, Emily Kukuk, Christian Furth, Paulina Cegla, Elia Lombardo, Joanna Kazmierska, Adrien Holzgreve, Iosif Strouthos, Carmen Stromberger, Claus Belka, Michael Baumann, Mechthild Krause, Guillaume Landry, Witold Cholewinski, Jorg Kotzerke, Daniel Zips, Jörg van den Hoff, Frank Hofheinz

**Affiliations:** 1 Department of Radiation Oncology, Charité– Universitätsmedizin Berlin, corporate member of Freie Universität Berlin, Humboldt-Universität zu Berlin, Berlin Institute of Health, Augustenburger Platz 1, 13353 Berlin, Germany; 2 Berlin Institute of Health at Charité, Universitätsmedizin Berlin, BIH Biomedical Innovation Academy, BIH Charité (Junior) Clinician Scientist Program, Berlin, Germany; 3 Department of Radiotherapy and Radiation Oncology, Faculty of Medicine and University Hospital Carl Gustav Carus, Technische Universität Dresden, Dresden, Germany; 4 German Cancer Consortium (DKTK), partner site Dresden, Germany, and German Cancer Research Center (DKFZ) Heidelberg, Heidelberg, Germany; 5 OncoRay– National Center for Radiation Research in Oncology, Faculty of Medicine and University Hospital Carl Gustav Carus, Technische Universität Dresden, Helmholtz-Zentrum Dresden– Rossendorf, Dresden, Germany; 6 Institute of Radiopharmaceutical Cancer Research, Helmholtz-Zentrum Dresden-Rossendorf, Dresden, Germany; 7 Department of Nuclear Medicine, Charité Universitätsmedizin Berlin, Freie Universität Berlin and Humboldt Universität zu Berlin, Berlin Institute of Health, Berlin, Germany; 8 Department of Nuclear Medicine, Greater Poland Cancer Centre, Poznan, Poland; 9 Department of Radiation Oncology, LMU University Hospital, LMU Munich, Munich, Germany; 10 Electroradiology Department, University of Medical Sciences, Poznan, Poland; 11 Radiotherapy Department II, Greater Poland Cancer Centre, Poznan, Poland; 12 Department of Nuclear Medicine, LMU University Hospital, LMU Munich, Munich, Germany; 13 Department of Radiation Oncology, German Oncology Center, European University Cyprus, Limassol, Cyprus; 14 Bavarian Cancer Research Center (BZKF), Munich, Germany; 15 German Cancer Consortium (DKTK), partner site Munich, a partnership between DKFZ and LMU University Hospital Munich, Munich, Germany; 16 Division of Radiooncology/Radiobiology, German Cancer Research Center (DKFZ), Heidelberg, Germany; 17 German Cancer Consortium (DKTK), partner site Munich, a partnership between DKFZ and LMU University Hospital Munich, Heidelberg, Germany; 18 Faculty of Medicine and University Hospital Carl Gustav Carus, Technische Universität Dresden, Dresden, Germany; 19 Helmholtz Association/Helmholtz-Zentrum Dresden - Rossendorf (HZDR), Dresden, Germany; 20 Institute of Radiooncology, Helmholtz-Zentrum Dresden-Rossendorf, Dresden, Germany; 21 Department of Nuclear Medicine, University Hospital Carl Gustav Carus, Technische Universität Dresden, Dresden, Germany; 22 German Cancer Consortium (DKTK), partner site Berlin, a partnership between DKFZ and Charité– Universitätsmedizin Berlin, Berlin, Germany; 23 National Tumor Center Berlin (NCT), Partner Site Berlin, Germany: German Cancer Research Center (DKFZ), Heidelberg, Germany; 24 Charité Comprehensive Cancer, Charité Universitätsmedizin Berlin, Berlin, Germany; 25 Berlin Institute of Health, Charité Universitätsmedizin Berlin, Berlin, Germany; 26 Max-Delbrück-Centrum für Molekulare Medizin, Helmholtz Association, Berlin, Germany

**Correction: Cancer Imaging**

10.1186/s40644-025-00836-6

Following publication of the original article [1], we were notified that the equal contribution note was missing: Sebastian Zschaeck and Marina Hajiyianni contributed equally.

The original article has been corrected.
