# Supplementary material for: Total lesion glycolysis of primary tumor and lymphnodes is a strong predictor for development of distant metastases in oropharyngeal carcinoma patients with independent validation in automatically delineated lesions
Source: Cancer Imaging. 2025 Feb 21;25:18. doi: 10.1186/s40644-025-00836-6 (PMC11844016; doi:10.1186/s40644-025-00836-6)

Total lesion glycolysis of primary tumor and lymphnodes is a strong predictor for development of distant metastases in oropharyngeal carcinoma patients with independent validation in automatically delineated lesions

**Supplementary Material**

**Supplementary methods 1:**

**Implementation of the CNN for automatic delineation**

The CNN was implemented using the Apache MXNet (version 1.9.0) package

for the R language and environment for statistical computing (version

4.2.0). Data preprocessing steps consisted of resampling of the PET

and CT images to a common voxel size of 2.5 × 2.5 × 2.5 mm and

cropping to a matrix size of 128 × 128 in the transaxial plane with

additional axial crop of head and neck region whenever

necessary. Consequently, CT intensity values were clipped to a range

of [−150, 150] HU and, both, PET and CT volumes were individually

normalized to the range [0, 1]. The network application process

consisted of predicting the probability maps for primary tumor and LN

metastases using five CNN models derived via 5-fold cross-validation

scheme. Prediction of the probability maps was preformed on partially

overlapping image subvolumes of the size of 128 × 128 × 32 which were

then recombined yielding the probability maps for the whole

volumes. Five sets of these probability maps originating from five CNN

models were averaged and the resulting class (background, primary

tumor, or LN metastasis) was assigned to each voxel according to the

highest of the probabilities. Finally, all ROIs with volumes < 0.1 ml

were removed.

**Supplementary table 1:** Explorative group: univariate Cox regression with respect to OS. Metric PET parameters (top) and binarized PET parameters (below).


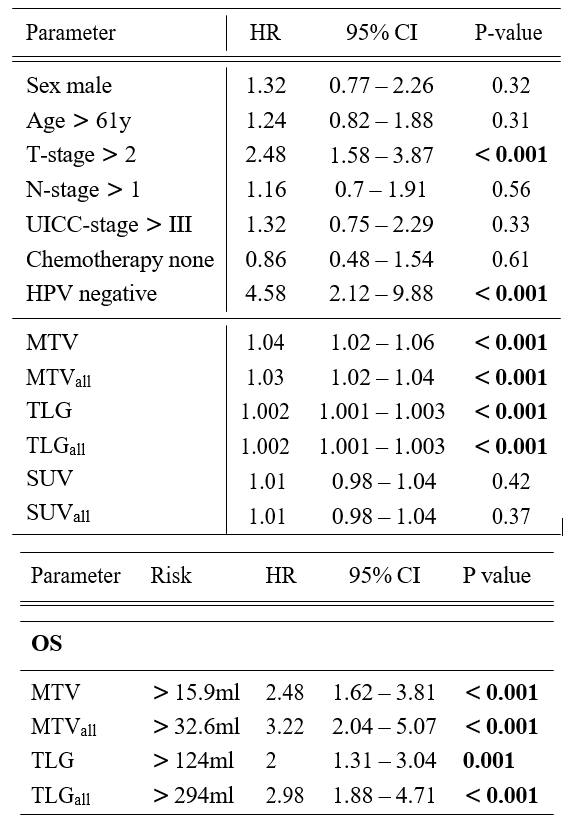


**Supplementary Figure 1:** Kaplan-Meier curves with respect to FFDM. Results for the validation group with manually delineated ROIs of both primary tumor (TLG_prim_) and primary tumor and lymphnodes (TLG_all_). Previously determined cutoffs were applied.


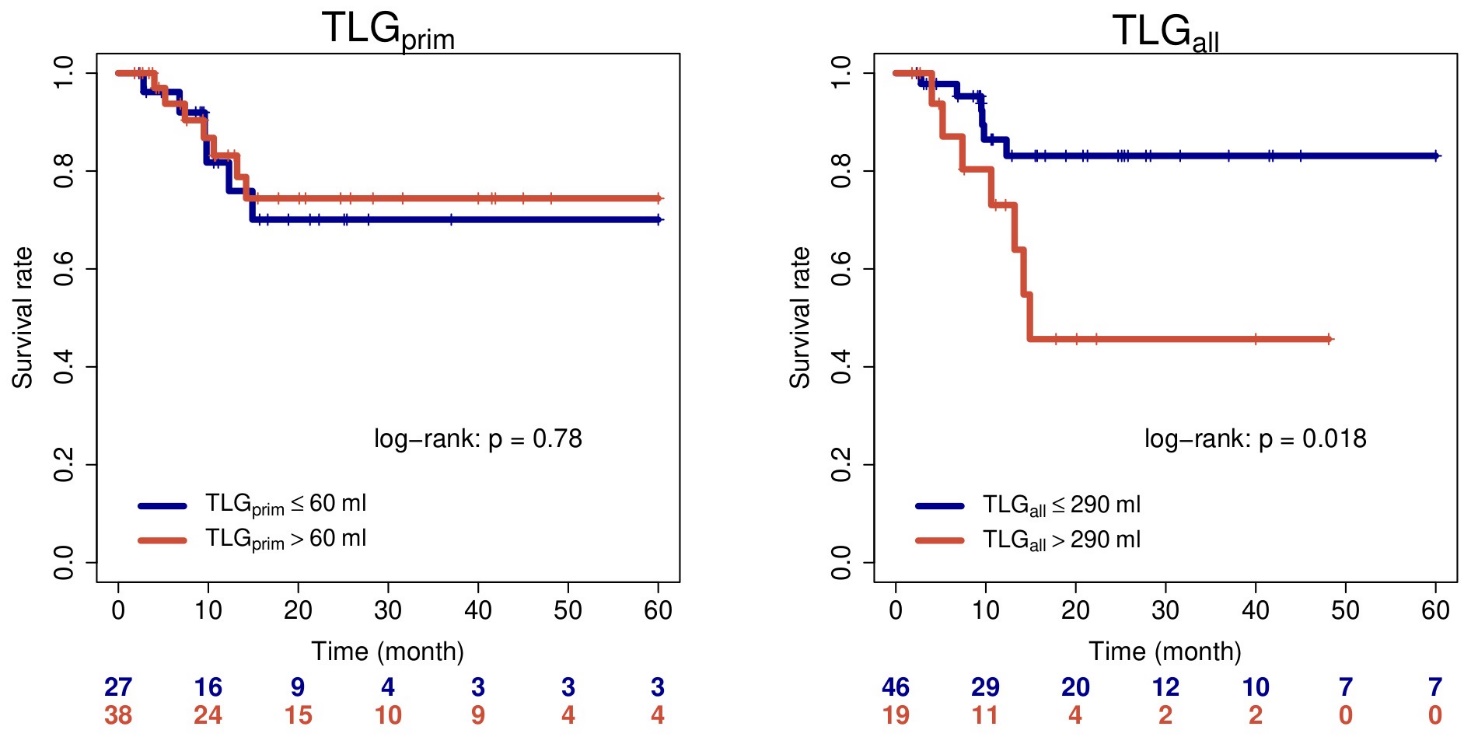


**Supplementary figure 2:** Kaplan Meier estimates with the endpoint FFDM in HPV positive patients.


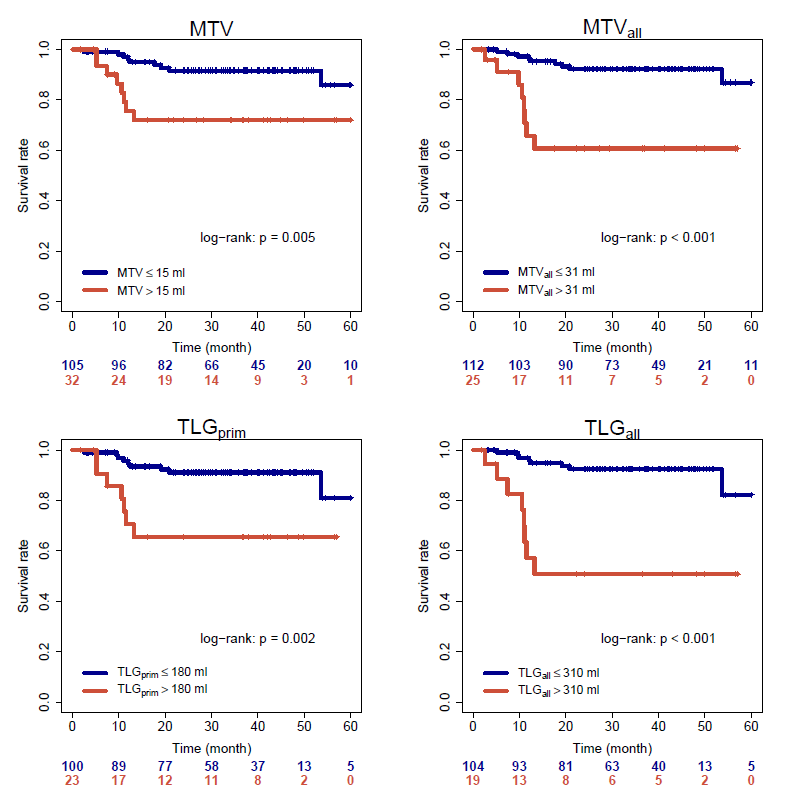


**Supplementary figure 3:** Kaplan Meier estimates with the endpoint FFDM in HPV negative patients.


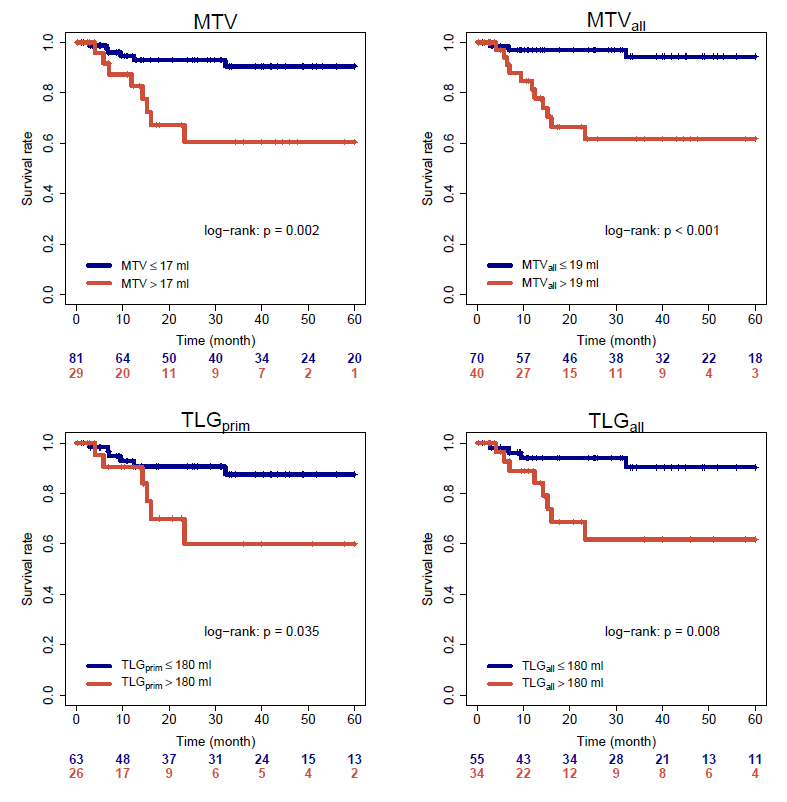

Supplement: Supplementary file 1 — Supplementary Material 1 [file 40644_2025_836_MOESM1_ESM.docx]
